# Multi-institutional retrospective study investigating blood culture protocols and test positivity in 701 dogs

**DOI:** 10.3389/fvets.2023.1301018

**Published:** 2023-12-11

**Authors:** Andrzej J. Ogrodny, Rinosh Mani, Sarah M. Schmid, Emily N. Gould, Claire L. Fellman, Ian DeStefano, Sarah Shropshire, Jillian M. Haines, Timothy A. Bolton, Sara A. Jablonski, Nicole Jess, Harry Cridge

**Affiliations:** ^1^Department of Small Animal Clinical Sciences, College of Veterinary Medicine, Michigan State University, East Lansing, MI, United States; ^2^Veterinary Diagnostic Laboratory, College of Veterinary Medicine, Michigan State University, Lansing, MI, United States; ^3^Department of Medical Sciences, College of Veterinary Medicine, University of Wisconsin-Madison, Madison, WI, United States; ^4^Department of Small Animal Clinical Sciences, College of Veterinary Medicine, University of Tennessee, Knoxville, TN, United States; ^5^Gastrointestinal Laboratory, Department of Small Animal Clinical Sciences, School of Veterinary Medicine & Biomedical Sciences, Texas A&M University, College Station, TX, United States; ^6^Department of Clinical Sciences, Cummings School of Veterinary Medicine, Tufts University, North Grafton, MA, United States; ^7^Department of Clinical Sciences, College of Veterinary Medicine & Biomedical Sciences, Colorado State University, Fort Collins, CO, United States; ^8^Department of Veterinary Clinical Sciences, College of Veterinary Medicine, Washington State University, Pullman, WA, United States; ^9^Department of Small Animal Clinical Sciences, College of Veterinary Medicine, Virginia Tech, Blacksburg, VA, United States; ^10^Center for Statistical Training and Consulting, Michigan State University, East Lansing, MI, United States

**Keywords:** bacteria, fungi, septicemia, susceptibility, MIC

## Abstract

**Objectives:**

(i) To determine the influence of specimen collection protocol (timing and specimen quantity), primary disease process, and pre-existing antimicrobial or immunosuppressive therapy on blood culture (BC) positivity and (ii) To determine agreement between urine culture and BC results.

**Animals:**

701 client-owned dogs.

**Methods:**

Multi-institutional retrospective study (2019–2022). Mixed-effect logistic regression was used to determine whether primary disease process, the number of BCs, or the timing of specimen collection was associated with BC positivity. Prediction plots were generated. Associations between urine culture and BC results were performed using logistic regression.

**Results:**

Dogs with a positive urine culture were more likely to have a positive BC (OR: 4.36, 95% CI: 2.12–8.97, *p* = 0.003). Dogs that had three BC specimens had the greatest odds of obtaining a positive BC result (adjusted predictive value: 0.44, 95% CI: 0.21–0.70), although this was not significant. Isolates from 38.5% of dogs with a positive BC had resistance to ≥3 antimicrobial classes. The timing between specimen collection had no significant association with BC positivity. Pre-existing antibiotic or immunosuppressive therapy had no significant association with BC positivity.

**Clinical relevance:**

Dogs with a positive urine culture were more likely to have a positive BC result.

## 1 Introduction

Dogs with bacteremia can develop sepsis which is associated with high morbidity and mortality (1–3). Blood cultures (BCs) are considered the gold standard diagnostic technique to identify and document bacteremia; however, they are expensive, and results may take several days. Advanced techniques such as matrix-assisted laser desorption ionization time of flight mass spectrometry (MALDI-TOF-MS) or polymerase chain reaction (PCR) have been proposed to reduce the timeline for identification of bacteremia, but these techniques do not provide information on *in vitro* antimicrobial susceptibility and do not replace the use of BCs in clinical practice (4, 5). Additionally, conflicting results exist regarding the utility of PCR to detect bacteremia (5, 6). The cost associated with performing serial BCs remains a major challenge and has led to variations in standard operating procedures, such as a reduction in specimens collected to reduce the total diagnostic cost (7, 8). It is unknown whether the number of BC-specimens is associated with the likelihood of obtaining a positive BC result in dogs. Fewer specimens might also make it more challenging to discern if a bacterial isolate is a contaminant or not. Additionally, the use of a single urine culture as a surrogate marker of bacteremia has also been proposed to reduce cost; however, the sensitivity for detection of a bacteremia from urine specimens was found to be poor (30%) in one study (9). An additional variation in BC protocols among clinicians is the timeline between collecting each specimen as part of a series of BCs. The impact of these protocol alterations on test positivity is unknown.

Thus, the objectives of our study were to determine the influence of specimen collection protocol (timing and specimen quantity), primary disease process, and pre-existing antimicrobial or immunosuppressive therapy on blood culture (BC) positivity. An additional objective was to determine agreement between urine culture and BC results.

## 2 Materials and methods

### 2.1 Case identification and data collection

Cases were identified by searching the medical record systems at 7 academic institutions for dogs that had BCs performed from Jan 1st, 2019, to December 31st, 2021. Medical and treatment records were analyzed, and the following data was extracted from each patient: BC isolate and *in vitro* susceptibility data, urine bacterial culture and susceptibility results, number of BCs, time between blood specimen collections, medical treatments (antimicrobials, immunosuppressive agents), and primary disease process. Species specific minimum inhibitory concentration (MIC) breakpoints were used where available, with human MIC breakpoints used if a species-specific MIC breakpoint was unavailable. Antimicrobial agents included any pre-existing treatment within 7 days of BC collection, or within 14 days if cefovecin was administered. The proportion of dogs that remained on antimicrobial treatment within 24 h of specimen collection was also recorded. Topical antimicrobials were not considered as part of pre-existing antimicrobial use. Immunosuppressive agent dosing thresholds were established for prednisone/prednisolone at >1 mg/kg/d (or equivalent dosing of dexamethasone). Chemotherapeutic agents were not considered as pre-existing immunosuppressive use. The time between blood specimen collections as part of a BC series were classified into the following groups: < 30 min, 30–60 min, 61–120 min, or > 120 min. Blood culture specimens were considered part of a single preplanned BC series (e.g., 1 multiple sample BC) when a regular repeating time interval was noted between specimen collections. When aerobic and anaerobic BCs were performed on the same specimen, they were classified as 1 BC-specimen. Contaminants were determined based on either criteria set by the reporting laboratory or review by a board-certified microbiologist, and these results were excluded from subsequent analysis. Primary disease processes were classified into the following groups based on complete medical record review by a single co-author (AJO): cardiac, dermatologic, gastrointestinal, hematology/immunology, hepatobiliary and pancreatic disease, musculoskeletal, neurologic, respiratory, reproductive/urogenital, and other. When animals had disease characteristics affecting multiple body systems, such as a dog with a urinary tract infection (UTI) and discospondylitis, the disease process that was suspected to have developed first (e.g., UTI) (based on medical record review) was listed as the primary disease process. When it was unknown which disease originated first, or when a systemic disease process was noted, the dog’s disease was listed in an alternate category, named “other.”

### 2.2 Data analysis

Signalment, primary disease process, and the presence or absence of concurrent antimicrobials or immunosuppressive agents were summarized in a descriptive manner. Mixed-effects logistic regression analysis were used to determine the effect of number of BC specimens collected and the timing of BC specimens on the likelihood of a positive BC result. Odds ratios were used to describe the predicted odds of BC positivity associated with each predictor in the model, with odds ratios greater than one indicating an increased odds of a positive result and odds ratios less than one indicating a decreased odds of a positive result. Primary disease process was investigated similarly. Institution was included as a random effect in the model. The reference population to which all others were compared was defined numerically and alphabetically by convention when a single population is not an obvious comparison group. Dogs that had a 1-specimen BC, and dogs with cardiac disease were therefore defined as the reference standards. Prediction plots were also generated to demonstrate the probability of a positive BC result by primary disease process, number of BCs performed, and time between BC specimens. The prediction plots were based on the results from the model and allow for visual comparisons between all groups, rather than only with the reference group. This did not rely on establishment of an arbitrary comparative group. Prediction plots were adjusted for the random effect of institution. Associations between urine culture results and the odds of a positive BC result were assessed using multiple logistic regression. All statistical analyses were performed by NJ using commercially available software (R Statistical Software v4.2.1, R Foundation for Statistical Computing, Vienna, Austria). The “stats” and “pROC” packages of R were utilized. *p* ≤ 0.05 were considered significant for all comparisons.

### 2.3 Methods for blood culture and susceptibility

BCs were performed using either VersaTREK (Remel Oxoid, Lenexa, KS) or BACTEC (BD Diagnostics, Sparks, MD) liquid BCs systems according to the manufacturer recommended protocols. In brief, each blood specimen was inoculated separately into an aerobic and anaerobic bottle and incubated at 37°C for 24 h. After the initial 24 h of incubation, the liquid media were analyzed using a Gram stain and subcultured onto standard agar plates (Colombia blood, chocolate, tryptic soy agar with 5% sheep blood, colistin nalidixic agar and MacConkey agar). If the culture remained negative, the BC bottles were incubated for a total of 5–7 days to confirm a lack of bacterial growth on standard agar media. Bacteria grown on agar plates were identified using Microflex LT MALDI ToF (Bruker, Billerica, MA) following manufacturer recommended protocols. Protocols for one study site differed in that separate aerobic and anerobic culture bottles were not used and inoculation to solid media was only performed if a positive signal/pressure change (VersaTREK system, TREK diagnostic Systems, Clevland, OH) was noted for the BC bottle during incubation or when Wampole isolator tubes were used (10). Antimicrobial susceptibility testing was done using broth microdilution method on Sensitive COMPGN1F (for gram negative bacteria) and COMPGP1F (for gram positive bacteria) (ThermoFisher, Cleveland, OH). Minimum inhibitory concentrations were determined, and susceptibility reported using CSLI guidelines (11–16). Antimicrobials evaluated are listed in Supplementary material 1.

### 2.4 Method of urine culture and susceptibility

Urine samples were inoculated on TSAB (trypticase soy agar with 5% sheep blood, Remel, Lenexa, KS) and two selective plates, MacConkey and CNA agar (Remel, Lenexa, KS). The plates were observed for bacterial growth after 24 h incubation at 37°C with 5% CO_2_. If no bacterial growth is observed the plates were re-incubated for another 2 days. Bacteria were then identified using Microflex LT MALDI-TOF (Bruker Daltonics, Bremen, Germany) following manufacturer recommended protocols. Antibiotic susceptibility testing was performed on isolated bacteria as described above.

## 3 Results

### 3.1 Animals

A total of 701 dogs were included in analysis. The median age of enrolled dogs was 7 years (range, 2 months – 16 years). There were 318 female (43 intact and 276 spayed females) and 380 were male (97 intact and 283 neutered males) dogs included. The sex of 2 dogs was not recorded. The most common dog breed was mixed breed (174 dogs), followed by Labrador retriever (61 dogs), German shepherd dog (55 dogs), golden retriever (38 dogs), boxer (16 dogs), border collie (12 dogs), mastiff (11 dogs), French bulldog (10 dogs), Siberian husky (10 dogs), greyhound (10 dogs), and Great Dane (10 dogs). Other breeds were represented by less than 10 dogs each. Weight was not recorded.

### 3.2 Bacterial culture and susceptibility results

One hundred and fifty-two dogs had positive BCs (excluding contaminants), yielding a 22% patient positivity rate. The most commonly isolated bacteria were *Staphylococcus* spp. [*Staphyloccus pseudintermedius* (34), *Staphylococcus aureus* (9) other *Staphylococcus* spp. (9)], *Streptococcus* spp. [Beta-hemolytic *Streptococcus* (20) and non-hemolytic *Streptococcus* (6)], and *Escherichia coli* (23). Other isolates identified included *Enterococcus* spp. (12), *Pasturella* spp. (11), *Actinomyces canis* (4), *Bacillus* spp. (4), *Pseudomonas aeruginosa* (3), and other bacterial species with <3 isolates identified (15). Of the pathogens cultured, 54 (38.5%) were found to have resistance to 3 or more antimicrobial classes. Antimicrobial susceptibility patterns are reported in Supplementary material 1.

### 3.3 Primary disease process

**Figure 1:**
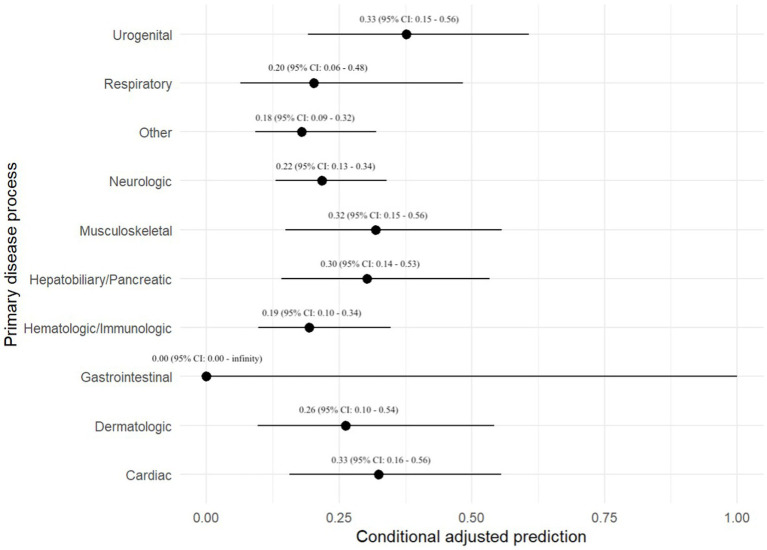
Prediction plot for odds of positive BC by primary disease process. This figure demonstrates a statistically modeled predictive value for BC positivity when compared to primary disease process. The black circle represents the predicted value, while the black line represents the 95% confidence interval.

The primary disease process was evaluated in all 701 dogs. The following disease processes were identified: neurologic disease in 142 (20.3%) dogs, hematologic or immunologic disease in 92 (13.1%), gastrointestinal disease in 72 (10.3%), musculoskeletal disease in 65 (9.3%), cardiac disease in 51 (7.3%), respiratory disease in 49 (7.0%), hepatobiliary or pancreatic disease in 45 (6.4%), urogenital disease in 42 (6.0%), and dermatologic disease in 31 (4.4%). One hundred and twelve dogs (16.0%) did not fit into any of the above categories. There was no association between primary disease process and the odds of a positive BC (Table 1). A prediction plot was also generated between primary disease process and odds of positive BC and no significant results were noted (Figure 1).

**Table 1:** Associations between primary disease process and odds of a positive BC.

Primary disease process	Odds ratio (95% CI)	*p*-value
Cardiac	N/A (reference population)	N/A (reference population)
Dermatologic	0.74 (0.18–2.99)	0.72
Gastrointestinal	0.00 (0.00–0.00)	1.00
Hematologic/ Immunologic	0.50 (0.17–1.49)	0.77
Hepatobiliary/ Pancreatic	0.90 (0.26–1.48)	0.63
Musculoskeletal	0.97 (0.28–3.33)	0.61
Neurologic	0.58 (0.22–1.48)	0.72
Other	0.45 (0.16–1.31)	0.79
Respiratory	0.53 (0.11–2.52)	0.81
Urogenital	1.26 (0.38–4.22)	0.50

This table demonstrates the odds ratio of BC positivity relative to the cardiac disease group.

### 3.4 Concurrent antimicrobials or immunosuppressive medications

Information on antimicrobial administration was available for 689 (98%) of dogs. Two-hundred and forty-seven (35.2%) dogs received antimicrobials within 7 days of BC collection and 3 dogs (0.4%) received cefovecin (off-label) within 14 days of BC collection. Two-hundred and forty-four (97.6%) of these dogs also received antimicrobials within 24 h of BC collection. Pre-existing antimicrobial therapy yielded an odds ratio of <1 for BC positivity (OR: 0.67, 95% CI: 0.31–1.46), and this relationship was not statistically significant (*p* = 0.65). One hundred dogs (14.3%) were receiving immunosuppressive medications at the time of specimen collection. There was no significant association between immunosuppressive therapy and odds of a positive BC (*p* = 0.74).

### 3.5 Number and timing of BC specimens

Eighty-nine (12.7%) dogs had a 1-specimen BC submitted, 243 (34.7%) dogs had a 2-specimen BC submitted, 85 (12.1%) dogs had a 3-specimen BC submitted, 256 (36.5%) dogs had a 4-specimen BC submitted, and 2 (< 1%) dogs had a 6-specimen BC submitted. The number of BCs submitted was unavailable for 26 dogs. There was no association between the number of BC specimens collected and the odds of a positive BC (Table 2).

**Table 2:** Associations between number of BC specimens and odds of a positive BC.

No. of blood specimens	Odds ratio (95% CI)	*p*-value
1	N/A (reference population)	N/A (reference population)
2	1.49 (0.51–4.33)	0.39
3	3.57 (1.00–12.75)	0.06
4	1.27 (0.43–3.75)	0.46
6	Excluded d/t sample size (2 dogs)	

This table demonstrates the odds ratio of BC positivity relative to the 1-specimen BC group.

**Figure 2:**
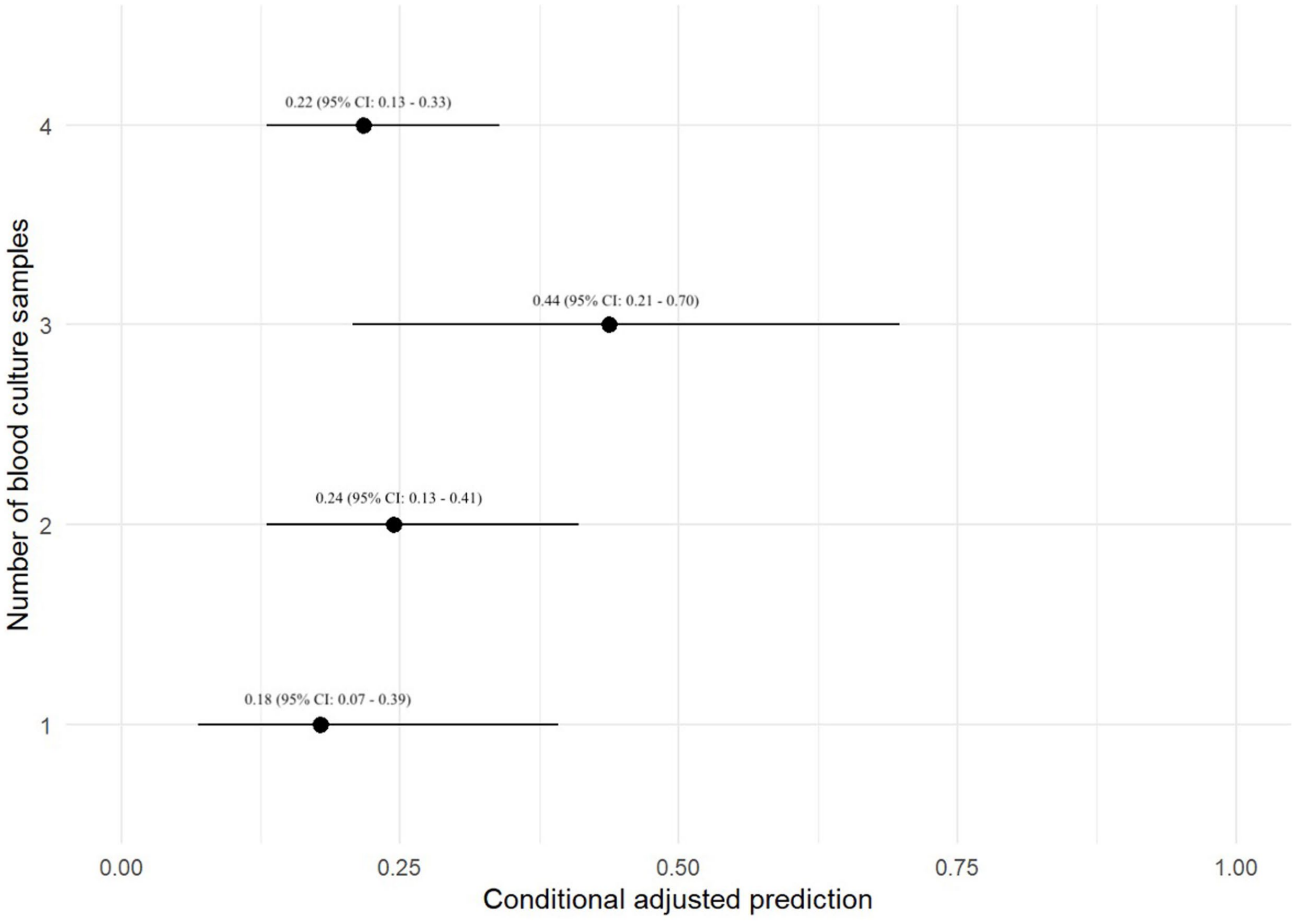
Prediction plot for odds of positive BC by numbers of BCs performed. This figure demonstrates a statistically modeled predictive value for BC positivity when compared to the number of cultures performed in each case. The black circle represents the predicted value, while the black line represents the 95% confidence interval.

A prediction plot was also generated between number of BC specimens performed and odds of a positive BC result, no significant results were noted (Figure 2). The group with 3-specimen BCs had the highest adjusted predictive value for BC positivity, but this was not significant (OR: 0.44, 95% CI: 0.21–0.70).

Three hundred and thirty-nine (48.4%) dogs had each of their BC specimens collected <30 min apart, 89 (12.7%) dogs had BCs collected 30–60 min apart, 90 (12.8%) dogs had BCs collected 61–120 min apart, and 38 (5.4%) dogs had BCs collected >120 min apart. The duration between BC collections were unknown in 56 (8.0%) dogs. The remaining 89 dogs (12.7%) only had a 1-specimen BC performed. The maximum duration between specimens was 8 h (*n* = 2). All other specimens were collected within 2–4 h of each other. There was no association between the timing between BC specimens and the odds of a positive BC (Table 3).

**Table 3:** Associations between timing between BC specimens and odds of a positive BC.

Time between BC specimens	Odds ratio (95% CI)	*p*-value
<30 min	N/A (reference population)	N/A (reference population)
30–60 min	0.43 (0.16–1.16)	0.80
61–120 min	1.02 (0.43–2.42)	0.51
>120 min	0.49 (0.14–1.68)	0.80

This table demonstrates the odds ratio of BC positivity relative to the time between collecting each blood sample, as part of a set of BCs.

**Figure 3:**
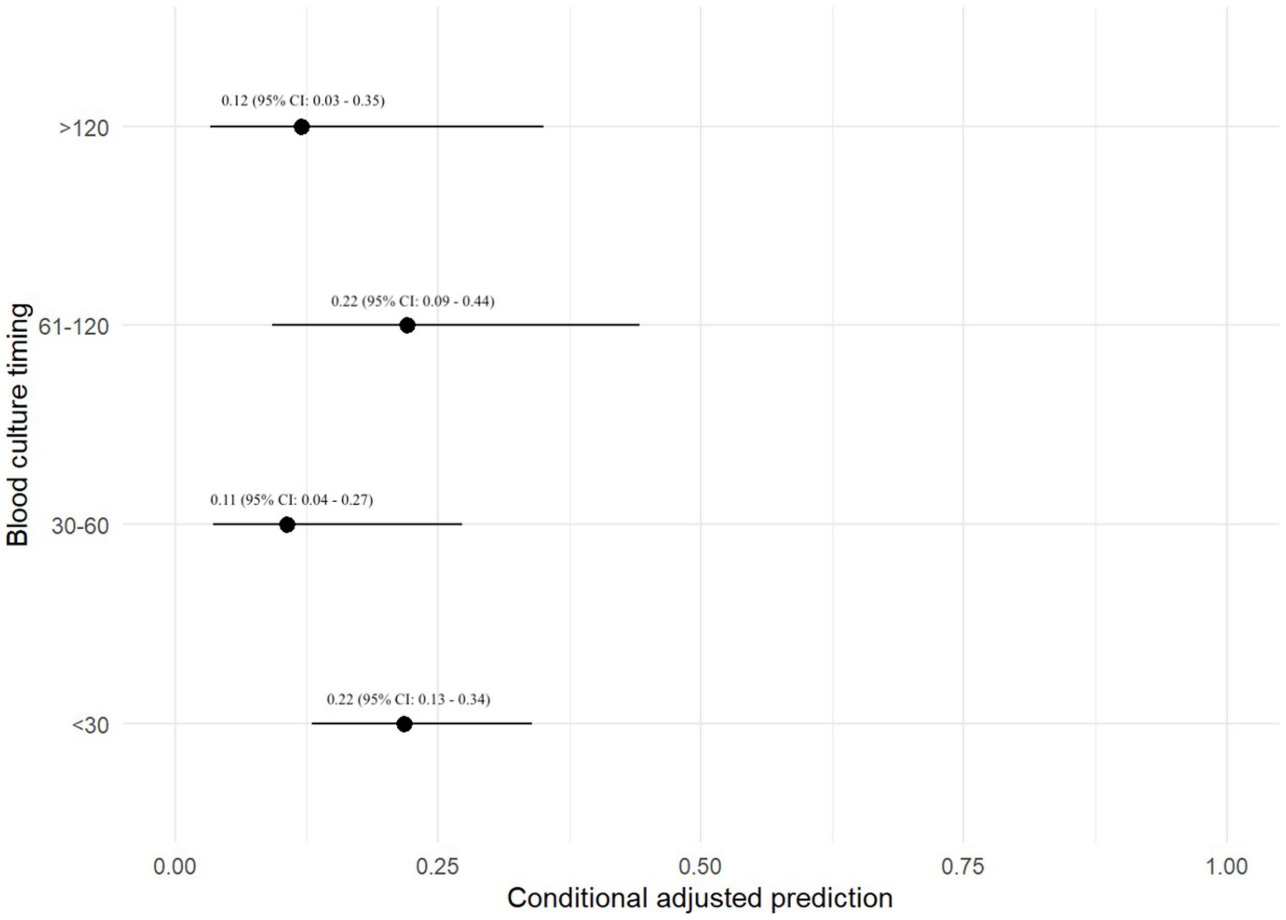
Prediction plot for odds of positive BC by BC timing. This figure demonstrates a statistically modeled predictive value for BC positivity when comparing the timespan between each blood sample as part of a set of BCs. The black circle represents the predicted value, while the black line represents the 95% confidence interval.

A prediction plot was also generated between timing between BC specimens and odds of a positive BC result (Figure 3). No significant results were found.

### 3.6 Associations between BC and urine culture results

Four hundred and ten dogs (58.5%) had a urine culture performed, 39 (9.5%) of which were positive (i.e., bacteria were cultured). Dogs that had a positive urine culture (*N* = 39) were significantly more likely to have a positive BC result than those that had no growth on a urine culture (OR: 4.36, 95% CI: 2.12–8.97, *p = 0*.003). Of the 701 dogs in the study, 28 had bacterial growth on both blood and urine cultures. Of those, isolates from 21/28 (75%) dogs were the same species of pathogen on both blood and urine culture. This is further summarized in Table 4.

**Table 4:** Summary of blood and urine culture findings.

No. of dogs that had a blood culture performed	No. of dogs with a positive blood culture result (i.e., bacterial growth)	No. of dogs that had a urine culture performed	No. of dogs with a positive urine culture (i.e., bacterial growth)	No. of dogs that had both a positive blood culture and a positive urine culture result
701	152/701 (21.7%)	410	39/410 (9.5%)	28/701 (4.0%)

This Table demonstrates the number of dogs that had blood and urine cultures performed and the results of these tests.

## 4 Discussion

This study evaluated factors associated with positive BC results in 701 dogs. The top 3 most common bacterial species identified were *Staphylococcus pseudintermedius*, *Escherichia coli*, and *beta-hemolytic Streptococcus* species. There was not a statistically significant relationship between primary disease process and odds of test positivity when utilizing a mixed-effect logistic regression analysis. There was also no statistically significant relationship between the number of BC specimens and odds of BC positivity when utilizing a mixed-effect logistic regression analysis; however, test positivity approached statistical significance when comparing dogs with a 3-specimen BC versus those with a 1-specimen BC. Additionally, the adjusted prediction plot of test positivity in dogs with a 3-specimen BC was 0.44, which was 1.8 times higher than the next closest group at 0.24 (2-specimen BC group). While we suspect that a 3-specimen BC protocol may balance cost and diagnostic yield, this hypothesis would need to be verified in large-scale prospective study. It is unclear why 4 BCs had a lower adjusted prediction for test positivity. In human medicine the number of BCs has been shown to influence the ability to detect bacteremia. Two BCs within 24 h are reported to detect ~90% of bloodstream infections in humans, whereas up to 4 BCs may be required to increase the detection rate to >99% (17). Single BCs have also been shown to be inferior to paired BCs for detection of bacterial pathogens in another study (18). Performing more than one BC is also of value when determining whether a bacterial isolate could be a contaminant. The timespan between each specimen collection as part of a set of BCs was also not significantly associated with BC positivity, and no major findings were detected utilizing predictive plots. The timespan between specimens in a set of BCs is therefore potentially of reduced importance, when compared to other variables such as number of blood specimens in a BC series.

Clinicians may also try to utilize case data to predict the diagnostic yield of BCs. Dogs with a positive urine culture had an increased odds of having a positive BC. In this study, isolates 21 of 28 dogs with concurrent growth on both blood and urine cultures were the same species of pathogen. This supports the findings of a previous study, in which 4 out of 5 dogs with a positive BC had a concurrently positive urine culture (19). However, it is important to note that some studies reveal that urine cultures have a low sensitivity for detection of concurrent bacteremia and a negative urine culture should not preclude a clinician from performing BCs (9). A reverse association may also be of clinical interest, but could not be evaluated in this study due to study design. Further investigation in the reverse direction is indicated in future studies.

In our study, pre-existing antimicrobial therapy did not have a significant effect on BC positivity, reinforcing that prior antimicrobial therapy should not preclude a clinician from performing BCs in a dog where bacteremia is of high clinical suspicion. Previous studies have also demonstrated that pre-existing antimicrobial therapy does not result in a lower percentage of positive BCs in dogs, and 32% of cats with positive BCs had been pre-treated with antimicrobials (20–22). This may be because bacteriostatic antimicrobials slow but fail to eliminate, bacterial growth in blood specimens (23). Antimicrobial resistance, dilution of antimicrobials to sub-therapeutic concentrations within the blood specimen, or clinician sampling biases may also be proposed explanations (20). It is also important to note that several antibiotics were used extra-label (“off label”) in this study including cefovecin. Extra-label drug use is very common in veterinary medicine in the United States as few drugs have been approved by the Federal Drug Administration for use in animals and for the broad range of medical conditions in which the medications are commonly used. The results of this study may however be impacted by population size and this should be considered when interpreting the results. In humans large scale studies have shown that collection of BCs after antimicrobial administration results in false negative BC results (24). The surviving sepsis campaign suggests that BCs should be obtained prior to starting antimicrobial therapy in humans with sepsis unless it results in a substantial delay in the start of antimicrobials (≥ 45 min) (25). Since specimen timing seemed less impactful in this study, the ability to collect cultures quickly (e.g., in less than 30 min) may facilitate collection prior to antimicrobial therapy and avoid delays in treatment for patients where sepsis is suspected. However future prospective studies are needed to confirm or refute this hypothesis. Immunosuppressive therapy had no effect on culture positivity in this study, although this may have been influenced by sample size and the different therapeutic regimes utilized.

The prevalence of dogs with positive BCs (22%) was similar to prior studies, and ~ 70% of isolates had resistance to at least one antimicrobial class. Although all institutions in our study were tertiary referral facilities, which likely impacted this finding, the high number of dogs with organisms resistant to greater than one antimicrobial reinforce the high number of dogs developing multi-drug resistant infections.

This study was limited by its retrospective nature, which inherently includes non-standardized diagnostic and therapeutic approaches. Although some institutions had a standard operating procedure for BCs in dogs, others did not, and it is impossible to determine the degree of compliance to such protocols retrospectively. Therefore, data such as specimen blood volume was not able to be accurately investigated in this study. In humans standard-volume (8.7 mL) BCs have a higher detection rate for bloodstream infection than low-volume (2.7 mL) cultures (26). This requires further investigation in dogs, and blood specimen volume may have influenced the results of this study. We recommend that blood volume is recorded on future BC results to assist in future studies and to act as a quality control. It is possible that sampling bias may have been inadvertently introduced by clinicians when determining BC protocols and this could influence the results of this study. Urine culture techniques were not standardized given the retrospective nature of the study and this could impact the results of the study. Efforts were made during study design and statistical methods to account for confounding variables, but it is possible that they exist and could influence the results of this study. Additionally the study population described here is focused on academic/tertiary referral centers and this may influence the results of the study. There is no standardized criteria for determination of blood culture contaminants in veterinary medicine, and as such our study design mimicked clinical practice by relying on interpretation by the laboratory or a board-certified microbiologist. Development and validation of such criteria would be of significant value but were outside the scope of this manuscript. Statistically non-significant results from this study should be interpreted with caution.

Importantly, this study assessed sampling protocols an BC results for a large population of dogs across multiple U.S. institutions. No significant relationship was found between the number or sample timing of BCs and culture positivity, although a positive urine culture was predictive of a positive BC. Future studies should seek to clarify the optimal specimen volumes and prospectively confirm the number of specimens to optimize BC cost effectiveness.

## Data availability statement

The raw data supporting the conclusions of this article will be made available by the authors, without undue reservation.

## Ethics statement

Ethical approval was not required for the studies involving animals in accordance with the local legislation and institutional requirements because not required – retrospective study. No patients or clients are identifiable. Written informed consent was not obtained from the owners for the participation of their animals in this study because not required – retrospective study. No patients or clients are identifiable.

## Author contributions

AO: Conceptualization, Formal analysis, Investigation, Methodology, Writing – original draft, Writing – review & editing, Data curation. RM: Conceptualization, Data curation, Formal analysis, Investigation, Methodology, Writing – original draft, Writing – review & editing, Supervision. SSc: Conceptualization, Data curation, Formal analysis, Investigation, Methodology, Writing – original draft, Writing – review & editing. EG: Conceptualization, Data curation, Formal analysis, Investigation, Methodology, Writing – original draft, Writing – review & editing. CF: Conceptualization, Data curation, Formal analysis, Investigation, Methodology, Writing – original draft, Writing – review & editing. ID: Conceptualization, Data curation, Formal analysis, Investigation, Methodology, Writing – original draft, Writing – review & editing. SSh: Conceptualization, Data curation, Formal analysis, Investigation, Methodology, Writing – original draft, Writing – review & editing. JH: Conceptualization, Data curation, Formal analysis, Investigation, Methodology, Writing – original draft, Writing – review & editing. TB: Conceptualization, Data curation, Formal analysis, Investigation, Methodology, Writing – original draft, Writing – review & editing. SJ: Conceptualization, Data curation, Formal analysis, Investigation, Methodology, Writing – original draft, Writing – review & editing. NJ: Conceptualization, Formal analysis, Investigation, Methodology, Writing – original draft, Writing – review & editing, Software. HC: Formal analysis, Investigation, Methodology, Writing – original draft, Writing – review & editing, Conceptualization, Supervision.
