# Supplementary material for: Multi-institutional retrospective study investigating blood culture protocols and test positivity in 701 dogs
Source: Front Vet Sci. 2023 Dec 11;10:1301018. doi: 10.3389/fvets.2023.1301018 (PMC10752604; doi:10.3389/fvets.2023.1301018)
Supplement: Supplementary file 1 [file Table_1.DOCX]

Supplementary Material 1

Multi-institutional retrospective study investigating blood culture protocols and test positivity in 701 dogs.

Andrzej J Ogrodny, Rinosh Mani, Sarah M. Schmid, Emily N Gould, Claire L. Fellman, Ian DeStefano, Sarah Shropshire, Jillian M. Haines, Timothy A. Bolton, Sara A. Jablonski, Nicole Jess, Harry Cridge^*^.

*** Correspondence:** Harry Cridge, MVB, MS, PG Cert Vet Ed, DACVIM (SAIM), DECVIM-CA, FHEA, MRCVS.
[harry.cridge@gmail.com](mailto:harry.cridge@gmail.com)

**Collated BC & Susceptibility Results**

| **Pathogen (Count)** | **Antimicrobial** | **Median MIC (Range)** | **Source of Break Point** | **% Susceptible** | **% Intermediate** | **% Resistant** |
| --- | --- | --- | --- | --- | --- | --- |
| *Actinomyces canis. (4)* | Penicillin | 0.12 (0.06 – 0.12) | Vet 06 1^st^ edition, 2017 | 4/4 (100%) | 0 | 0 |
|  | Cefazolin | 1.00 (1.00– 1.00) | Vet 06 1^st^ edition, 2017 | 4/4 (100%) | 0 | 0 |
|  | Amikacin | 4.00 (2.00 – 8.00) | M 45 3^rd^ edition, 2015 | 4/4 (100%) | 0 | 0 |
|  | Gentamycin | 1.00 (1.00 – 1.00) | Vet 06 1^st^ edition, 2017 | 4/4 (100%) | 0 | 0 |
|  | Clindamycin | 0.50 (0.50 – 0.50) | Vet 06 1^st^ edition, 2017 | 4/4 (100%) | 0 | 0 |
|  | Erythromycin | 0.50 (0.50 – 0.50) | Vet 06 1^st^ edition, 2017 | 4/4 (100%) | 0 | 0 |
|  | Doxycycline | 2.00 (2.00 – 16.00) | Vet 06 1^st^ edition, 2017 | 3/4 (75%) | 0 | 1/4 (25%) |
|  | Trimethoprim/Sulpha | 0.50/0.50 (0.50 – 0.50, 0.50-0.50) | Vet 06 1^st^ edition, 2017 | 4/4 (100%) | 0 | 0 |
|  | Chloramphenicol | 4.00 (4.00 – 4.00) | Vet 06 1^st^ edition, 2017 | 4/4 (100%) | 0 | 0 |
| *Bacillus spp.* (4) | Ampicillin | 0.25 (0.25 – 0.25) | Vet 06 1^st^ edition, 2017 | 4/4 (100%) | 0 | 0 |
|  | Penicillin | 0.06 (0.06 – 0.06) | Vet 06 1^st^ edition, 2017 | 4/4 (100%) | 0 | 0 |
|  | Cefazolin | 2.00 (2.00 – 2.00) | Vet 06 1^st^ edition, 2017 | 4/4 (100%) | 0 | 0 |
|  | Amikacin | 16.00 (16.00 – 16.00) | Vet 06 1^st^ edition, 2017 | 4/4 (100%) | 0 | 0 |
|  | Gentamycin | 4.00 (4.00 – 4.00) | Vet 06 1^st^ edition, 2017 | 4/4 (100%) | 0 | 0 |
|  | Clindamycin | 1.00 (1.00 – 1.00) | Vet 06 1^st^ edition, 2017 | 0 | 4/4 (100%) | 0 |
|  | Erythromycin | 0.25 (0.25 – 0.25) | Vet 06 1^st^ edition, 2017 | 4/4 (100%) | 0 | 0 |
|  | Doxycycline | 0.12 (0.12 – 0.12) | Vet 06 1^st^ edition, 2017 | 4/4 (100%) | 0 | 0 |
|  | Tetracycline | 0.25 (0.25 – 0.25) | Vet 06 1^st^ edition, 2017 | 4/4 (100%) | 0 | 0 |
|  | Trimethoprim/Sulpha | 2.00/38.00 (2.00-2.00, 38.00-38.00) | Vet 06 1^st^ edition, 2017 | 4/4 (100%) | 0 | 0 |
|  | Chloramphenicol | 8.00 (8.00 – 8.00) | Vet 06 1^st^ edition, 2017 | 4/4 (100%) | 0 | 0 |
|  | Rifampin | 1.00 (1.00 – 1.00) | Vet 06 1^st^ edition, 2017 | 4/4 (100%) | 0 | 0 |
| *Escherichia coli (23)* | Ampicillin | 32.00 (2.00 -32.01) | Vet 08 4^th^ edition, 2018 | 0 | 0 | 21/21 (100%) |
|  | Amoxicillin/CA | 8.00 (0.25 – 32.00) | Vet 08 4^th^ edition, 2018 | 0 | 0 | 23/23 (100%) |
|  | Piperacillin/Tazobactam | 4.00, 4.00 (4.00 – 4.00, 4.00-64.00) | Vet 01 5^th^ edition, 2020 | 13/14 (93%) | 0 | 1/14 (7%) |
|  | Cefalexin | 4.00 (4.00 – 64.00) | Vet 01 5^th^ edition, 2020 | 20/20 (100%) | 0 | 0 |
|  | Cefpodoxime | 1.00 (0.25 – 8.00) | Vet 08 4^th^ edition, 2018 | 11/18 (61%) | 0 | 7/18 (39%) |
|  | Ceftazidime | 0.50 (0.12 – 32.00) | M100 32^nd^ edition, 2022 | 19/22 (86%) | 1/22 (5%) | 2/22 |
|  | Imipenem | 0.25 (0.25 – 0.25) | M100 32^nd^ edition, 2022 | 16/16 (100% | 0 | 0 |
|  | Amikacin | 2.00 (1.99 – 8.00) | Vet 08 4^th^ edition, 2018 | 22/23 (96%) | 1/23 (4%) | 0 |
|  | Gentamycin | 1.00 (0.5-2.00) | Vet 01 5^th^ edition, 2020 | 23/23 (100%) | 0 | 0 |
|  | Doxycycline | 5.00 (0.50 – 16.00) | M100 32^nd^ edition, 2022 | 8/16 (50%) | 1/16 (6%) | 7/16 (44%) |
|  | Tetracycline | 1.00 (1.00-16.00) | Vet 01 5^th^ edition, 2020 | 10/13 (77%) | 0 | 3/13 (23%) |
|  | Enrofloxacin | 0.12 (0.12 – 4.00) | Vet 01 5^th^ edition, 2020 | 17/22 (77%) | 1/22 (5%) | 4/22 (18%) |
|  | Marbofloxacin | 0.50 (0.12 - 4.00) | Vet 01 5^th^ edition, 2020 | 18/23 (78%) | 0 | 5/23 (22%) |
|  | Pradofloxacin | 0.25 (0.25 - 0.25) | Vet 08 4^th^ edition, 2018 | 5/5 (100%) | 0 | 0 |
|  | Trimethoprim/Sulpha | 20.00/20.00 (0.05-320.00, 9.50-32.00) | M100 32^nd^ edition, 2022 | 21/23 (91%) | 0 | 2/23 (9%) |
|  | Chloramphenicol | 4.00 (2.00 - 64.00 ) | M100 32^nd^ edition, 2022 | 16/23 (65%) | 6/23 (26%) | 1/23 (9%) |
| *Enterococcus spp. (12)* | Penicillin | 4.00 (0.50 – 64.00) | M100 32^nd^ edition, 2022 | 8/11 (73%) | 0 | 3/11 (27%) |
|  | Erythromycin | 8.00 (0.25-8.00) | M100 32^nd^ edition, 2022 | 1/10 (10%) | 3/10 (30%) | 6/10 (60%) |
|  | Doxycycline | 0.50 (0.50 – 16.00) | M100 32^nd^ edition, 2022 | 6/9 (67%) | 0 | 3/9 (33%) |
|  | Minocycline | 0.50 (0.50 -16.00) | M100 32^nd^ edition, 2022 | 6/8 (75%) | 0 | 2/6 (25%) |
|  | Enrofloxacin | 2.00 (0.50 – 4.00) | Vet 01 5^th^ edition, 2020 | 3/11 (28%) | 4/11 (36%) | 4/11 (36%) |
|  | Marbofloxacin | 3.00 (2.00 – 4.01 ) | Vet 01 5^th^ edition, 2020 | 0 | 5/11 (45%) | 6/11 (55%) |
|  | Chloramphenicol | 8.00 (4.00 – 32.00) | M100 32^nd^ edition, 2022 | 11/12 (92%) | 0 | 1/11 (8%) |
| *Pasteurella spp. (11)* | Ampicillin | 8.01 (0.12 – 8.01) | Vet 08 4^th^ edition, 2018 | 10/10 (100% | 0 | 0 |
|  | Amoxicillin/CA | 8.01 (0.25 – 8.01) | Vet 08 4^th^ edition, 2018 | 10/10 (100%) | 0 | 0 |
|  | Cefazolin | 1.00 (1.00 -1.00) | Vet 08 4^th^ edition, 2018 | 10/10 (100%) | 0 | 0 |
|  | Cefpodoxime | 2.00 (2.00 – 2.00) | Vet 08 4^th^ edition, 2018 | 10/10 (100%) | 0 | 0 |
|  | Doxycycline | 2.00 (0.25 – 2.00) | M 45 3^rd^ edition, 2015 | 9/9 (100%) | 0 | 0 |
|  | Enrofloxacin | 0.25 (0.12 – 0.25) | Vet 06 1^st^ edition, 2017 | 9/9 (100%) | 0 | 0 |
| *Pseudomonas aeruginosa (3)* | Piperacillin/Tazobactam | 8.00 (8.00-16.00) | M100 32^nd^ edition, 2022 | 3/3 (100%) | 0 | 0 |
|  | Cefpodoxime | 8.01 (2.00 – 8.01) | Vet 01 5^th^ edition, 2020 | 0 | 0 | 3/3 (100%) |
|  | Ceftazidime | 4.00 (4.00 – 4.00) | Vet 01 5^th^ edition, 2020 | 3/3 (100%) | 0 | 0 |
|  | Amikacin | 4.00 (3.99 – 8.00) | M100 32^nd^ edition, 2022 | 3/3 (100%) | 0 | 0 |
|  | Gentamycin | 1.00 (1.00 – 2.00) | Vet 01 5^th^ edition, 2020 | 3/3 (100%) | 0 | 0 |
|  | Enrofloxacin | 1.00 (1.00 – 2.00) | Vet 01 5^th^ edition, 2020 | 0 | 3/3 (100%) | 0 |
|  | Marbofloxacin | 0.50 (0.25 – 1.00) | Vet 01 5^th^ edition, 2020 | 3/3 (100%) | 0 | 0 |
|  | Trimethoprim/Sulpha | 4.00/4.00 (4.00 – 4.0, 4.00 – 4.00) | M100 32^nd^ edition, 2022 | 0 | 0 | 3/3 (100%) |
| *Beta-hemolytic Streptococcus (20)* | Ampicillin | 0.25 (0.12 -- 0.25) | Vet 08 4^th^ edition, 2018 | 10/10 (100%) | 0 | 0 |
|  | Penicillin | 0.06 (0.05 – 0.06) | Vet 08 4^th^ edition, 2018 | 10/10 (100%) | 0 | 0 |
|  | Amoxicillin/CA | 0.25 (0.12 – 0.25) | Vet 01 5^th^ edition, 2020 | 11/11 (100%) | 0 | 0 |
|  | Cefazolin | 1.50 (1.00 – 2.00) | Vet 01 5^th^ edition, 2020 | 12/12 (100%) | 0 | 0 |
|  | Cefovecin | 0.06 (0.06 – 0.60) | Vet 01 5^th^ edition, 2020 | 6/6 (100%) | 0 | 0 |
|  | Cefoxitin | 2.00 (2.00 – 2.00) | Vet 01 5^th^ edition, 2020 | 12/12 (100%) | 0 | 0 |
|  | Cefpodoxime | 2.00 (2.00 – 2.00) | Vet 01 5^th^ edition, 2020 | 12/12 (100%) | 0 | 0 |
|  | Clindamycin | 0.50 (0.50 – 0.50) | Vet 01 5^th^ edition, 2020 | 13/13 (100%) | 0 | 0 |
|  | Doxycycline | 0.25 (0.12 – 8.00) | Vet 01 5^th^ edition, 2020 | 6/11 (55%) | 0 | 5/11 (45%) |
|  | Enrofloxacin | 0.50 (0.50 – 1.00) | Vet 01 5^th^ edition, 2020 | 9/12 (75%) | 3/12 (25%) | 0 |
|  | Marbofloxacin | 1.00 (1.00 -2.00) | Vet 01 5^th^ edition, 2020 | 10/12 (83%) | 2/12 (17%) | 0 |
| *Staphylococcus pseudintermedius (34)* | Ampicillin | 0.50 (0.12-8.01) | Vet 01 5^th^ edition, 2020 | 24/31 (7%) | 0 | 7/31 (23%) |
|  | Oxacillin | 0.25 (0.25-4.00) | Vet 01 5^th^ edition, 2020 | 22/28 (79%) | 0 | 6/28 (21%) |
|  | Cefpodoxime | 0.50 (0.50 – 8.00) | Vet 01 5^th^ edition, 2020 | 30/34 (88%) | 0 | 4/34 (12%) |
|  | Amikacin | 2.00 (2.00-16.00) | Vet 01 5^th^ edition, 2020 | 29/33 (88%) | 0 | 4/33 (12%) |
|  | Gentamicin | 0.50 (0.50 – 16.00) | Vet 01 5^th^ edition, 2020 | 27/34 (79%) | 4/34 (12%) | 3/34 (9%) |
|  | Doxycycline | 2.00 (0.12 – 8.00) | Vet 01 5^th^ edition, 2020 | 20/33 (61%) | 0 | 13/33 (39%) |
|  | Erythromycin | 0.25 (0.25 – 8.00) | Vet 01 5^th^ edition, 2020 | 21/32 (66%) | 0 | 11/32 (34%) |
|  | Enrofloxacin | 0.50 (0.25 – 4.00) | Vet 01 5^th^ edition, 2020 | 24/32 (75%) | 0 | 8/32 (25%) |
|  | Marbofloxacin | 0.50 (0.50 – 4.00) | Vet 01 5^th^ edition, 2020 | 26/33 (79%) | 0 | 7/33 (21%) |
|  | Pradofloxacin | 0.12 (0.12 – 4.00) | Vet 01 5^th^ edition, 2020 | 18/22 (82%) | 0 | 4/22 (18% |
|  | Chloramphenicol | 8.00 (4.00 -64.00) | Vet 01 5^th^ edition, 2020 | 29/33 (88%) | 0 | 4/33 (12%) |
| *Staphylococcus aureus (9)* | Ampicillin | 0.25 (0.25 – 0.25) | Vet 01 5^th^ edition, 2020 | 3/8 (38%) | 0 | 5/8 (62%) |
|  | Amoxicillin/CA | 0.25, 0.12 (0.25 – 2.00, 0.12 – 0.12) | Vet 01 5^th^ edition, 2020 | 3/9 (33%) | 1/9 (11%) | 5/9 (56%) |
|  | Cefalexin | 2.00 (2.00 –2.00) | Vet 01 5^th^ edition, 2020 | 5/5 (100%) | 0 | 0 |
|  | Cefovecin | 0.50 (0.50 – 0.50) | Vet 01 5^th^ edition, 2020 | 5/7 (72%) | 2/7 (28%) | 0 |
|  | Cefpodoxime | 0.50 (0.50 – 2.00) | Vet 01 5^th^ edition, 2020 | 8/8 (100%) | 0 | 0 |
|  | Amikacin | 0.50 (0.50 – 2.00) | Vet 01 5^th^ edition, 2020 | 7/7 (100%) | 0 | 0 |
|  | Gentamicin | 1.00 (0.50 – 4.00) | Vet 01 5^th^ edition, 2020 | 8/8 (100%) | 0 | 0 |
|  | Clindamycin | 0.25 (0.25 – 5.00) | Vet 01 5^th^ edition, 2020 | 9/9 (100%) | 0 | 0 |
|  | Erythromycin | 0.25 (0.25 – 4.00) | Vet 01 5^th^ edition, 2020 | 8/9 (89%) | 0 | 1/9 (11%) |
|  | Doxycycline | 2.00 (0.25 – 2.00) | Vet 01 5^th^ edition, 2020 | 2/9 (22%) | 2/9 (22%) | 5/9 (56%) |
|  | Marbofloxacin | 1.00 (1.00 – 2.00) | Vet 01 5^th^ edition, 2020 | 8/9 (89%) | 1/9 (11/%) | 0 |
|  | Trimethoprim/Sulpha | 0.50, 9.50 (0.25 – 10.00, 0.25 – 9.50) | Vet 01 5^th^ edition, 2020 | 8/8 (100%) | 0 | 0 |
|  | Chloramphenicol | 8.00 (4.00 -- 8.00) | Vet 01 5^th^ edition, 2020 | 8/8 (100%) | 0 | 0 |

Supplementary Material 1 represents the bacterial isolate susceptibility patterns of the etiologic agents identified in this study. Note that suspected contaminants (n = 16), bacterial species with < 3 isolates identified (n=15), non-beta hemolytic *Streptococcus* spp. (n=8), and *Staphylococcus* spp. that are not *S. aureus* (n=9) have been excluded from this table.
